# A retrospective analysis of the relationship between anti-cyclic citrullinated peptide antibody and the effectiveness of abatacept in rheumatoid arthritis patients

**DOI:** 10.1038/s41598-020-76842-4

**Published:** 2020-11-12

**Authors:** Daihei Kida, Nobunori Takahashi, Atsushi Kaneko, Yuji Hirano, Takayoshi Fujibayashi, Yasuhide Kanayama, Masahiro Hanabayashi, Yuichiro Yabe, Hideki Takagi, Takeshi Oguchi, Takefumi Kato, Koji Funahashi, Takuya Matsumoto, Masahiko Ando, Yachiyo Kuwatsuka, Eiichi Tanaka, Hidekata Yasuoka, Yuko Kaneko, Shintaro Hirata, Kosaku Murakami, Yasumori Sobue, Tsuyoshi Nishiume, Mochihito Suzuki, Yutaka Yokota, Kenya Terabe, Shuji Asai, Naoki Ishiguro, Toshihisa Kojima

**Affiliations:** 1 Department of Orthopedic Surgery and Rheumatology, Nagoya Medical Center, 4-1-1 Sanno-maru, Naka-ku, Nagoya, Aichi Japan; 2 Department of Orthopedic Surgery and Rheumatology, Nagoya University Hospital, Nagoya University Graduate School of Medicine, 65 Tsurumai-cho, Showa-ku, Nagoya, Aichi 466-8550 Japan; 3 Department of Rheumatology, Toyohashi Municipal Hospital, 50 Hakken-nishi, Aotake-cho, Toyohashi, Japan; 4 Department of Orthopedic Surgery, Konan Kosei Hospital, 137 Oomatsubara, Takaya-cho, Konan, Aichi Japan; 5 Department of Orthopedic Surgery, Toyota Kosei Hospital, 500-1 Ibohara, Josui-cho, Toyota, Aichi Japan; 6 Department of Orthopedic Surgery, Ichinomiya Municipal Hospital, 2-2-22 Bunkyo, Ichinomiya, Aichi Japan; 7 Department of Rheumatology, Tokyo Shinjuku Medical Center, 5-1 Tsukudo-cho, Shinjuku-ku, Tokyo, Japan; 8 Department of Orthopedic Surgery, Nagoya Central Hospital, 3-7-7 Taiko, Nakamura-ku, Nagoya, Aichi Japan; 9 Department of Orthopedic Surgery, Anjo Kosei Hospital, 28 Higashihirokute, Anjo, Aichi Japan; 10 Kato Orthopedic Clinic, 8-4 Minami-myoudaiji-cho, Okazaki, Aichi Japan; 11 Department of Orthopedic Surgery, Kariya-Toyota General Hospital, 5-15 Sumiyoshi-cho, Kariya, Aichi Japan; 12 Department of Orthopedic Surgery, Shizuoka Kosei Hospital, 23 Kitaban-cho, Aoi-ku, Shizuoka, Japan; 13 Department of Advanced Medicine, Nagoya University Hospital, 65 Tsurumai-cho, Showa-ku, Nagoya, Aichi Japan; 14 Department of Rheumatology, School of Medicine, Tokyo Women’s Medical University, 8-1 Kawada-cho, Shinjuku-ku, Tokyo, Japan; 15 Division of Rheumatology, Department of Internal Medicine, Fujita Health University School of Medicine, 1-98 Dengakugakubo, Kutsukake-cho, Toyoake, Aichi Japan; 16 Department of Internal Medicine, Keio University Hospital, 35 Shinanomachi, Shinjuku-ku, Tokyo, Japan; 17 Department of Clinical Immunology and Rheumatology, Hiroshima University Hospital, 1-2-3 Kasumi, Minami-ku, Hiroshima, Japan; 18 Department of Rheumatology and Clinical Immunology, Kyoto University Graduate School of Medicine, 54 Kawaharacho, Shogoin, Sakyo-ku, Kyoto, Japan

**Keywords:** Rheumatoid arthritis, Outcomes research

## Abstract

This study aimed to evaluate the effectiveness of abatacept (ABA) by anti-cyclic citrullinated peptide (ACPA) status on disease activity as well as radiographic progression in patients with rheumatoid arthritis (RA) in clinical settings. A retrospective cohort study was conducted using data from a multicenter registry. Data from a total of 553 consecutive RA patients treated with intravenous ABA were included. We primarily compared the status of disease activity (SDAI) and radiographic progression (van der Heijde modified total Sharp score: mTSS) between the ACPA-negative (N = 107) and ACPA-positive (N = 446) groups. ‘ACPA positive’ was defined as ≥ 13.5 U/mL of anti-CCP antibody. Baseline characteristics between groups were similar. The proportion of patients who achieved low disease activity (LDA; SDAI ≤ 11) at 52 weeks was significantly higher in the ACPA-positive group. Multivariate logistic regression analysis identified ACPA positivity as an independent predictor for achievement of LDA at 52 weeks. Drug retention rate at 52 weeks estimated by the Kaplan–Meier curve was significantly higher in the ACPA-positive group. Achievement rate of structural remission (ΔmTSS ≤ 0.5) at 52 weeks was similar between groups. ABA treatment demonstrated a significantly higher clinical response and higher drug retention rate in ACPA-positive patients. Progression of joint destruction was similar between the ACPA-negative and ACPA-positive groups. Close attention should be paid to joint destruction even in patients showing a favorable response to ABA, especially when the ACPA status is positive.

## Introduction

Abatacept (ABA) is the first and only one of biological disease-modifying anti-rheumatic drugs (bDMARDs) for rheumatoid arthritis (RA) that inhibits T lymphocyte activation by binding to CD80/86, thereby modulating its interaction with CD28. Clinical outcomes of ABA have been reported in several randomized controlled trials (RCTs)^1,2^ and in clinical practice^3,4^. We also previously published several reports describing the clinical effectiveness and safety profile of ABA in routine clinical practice using data from a Japanese multicenter registry^5–7^. However, we did not initially collect anti-cyclic citrullinated peptide antibody (ACPA) [most commonly measured by anti-cyclic citrullinated peptide (anti-CCP)] status data from all registered patients^8^. Since the ACPA status has been reported to be associated with clinical response and drug retention of ABA from several groups^3,9,10^, we again investigated the unregistered data for ACPA status from all cases.

Gottenberg et al. reported for the first time that ACPA positivity predicted a good response to ABA and higher retention rate of ABA treatment using the data from the French Orencia and Rheumatoid Arthritis (ORA) registry^3^. Nusslein et al. reported that ACPA positivity predicted a higher retention rate of ABA^9^. Sokolove et al. reported that ACPA positivity was associated with a better response to ABA as a sub-analysis of the Abatacept versus adaliMumab comParison in bioLogic-naïvE RA subjects with background MTX (AMPLE) study^10,11^. Taken together, these results from real-world data and a clinical trial, a better response to ABA treatment and higher retention rate of ABA seem to be associated with ACPA positivity. However, only few reports described the association between detailed change in disease activity and ACPA positivity. Additionally, data on the association between progression of joint destruction and ACPA positivity in RA patients when treated with ABA are scarce at this time^12–15^. An investigation of the progression of joint destruction in ACPA-positive RA patients treated with ABA in daily clinical practice would be informative.

In this study, we compared change in disease activity over time, and achievement rates of low disease activity and radiographic remission at 52 weeks between ACPA-negative and ACPA-positive RA patients treated with ABA using data from a multicenter registry system.

## Materials and methods

### A multicentre registry system for RA patients treated with bDMARDs

All eligible patients were registered in and followed by the Tsurumai Biologics Communication Registry (TBCR). The TBCR is a registry of patients with RA receiving treatment with biologics. The registry began in 2008 and was developed to analyze the long-term prognosis of patients undergoing treatment with biologics in clinical practice^5,16^. Data were collected prospectively beginning in 2008, as well as retrospectively for patients who had been treated with biologics through 2008. All 2827 patients registered in the TBCR as of April 2015 met the 1987 American College of Rheumatology (ACR) or the 2010 ACR/ European League Against Rheumatism (EULAR) classification criteria for RA^17^. Information on medication history was collected at clinic visits to TBCR-affiliated institutions. Registry data are updated once per year and include information on drug continuation, reasons for discontinuation (e.g., insufficient effectiveness), and adverse events. Patient anonymity was maintained during data collection and security of personal information was strictly controlled. This study was approved by the Nagoya University Graduate School of Medicine Ethics Committee (Approval No.: 2011-1164). Written informed consent was obtained from all participants of this study. All methods were carried out in accordance with relevant guidelines and regulations.

### Current retrospective study

This study was conducted to compare SDAI status and radiographic progression between ACPA-positive and ACPA-negative RA patients treated with ABA. We collected data again for this retrospective study, focusing on unregistered data regarding ACPA status. The present study included 554 consecutive patients with ACPA data who were treated with intravenous (IV) ABA and prospectively observed for longer than 52 weeks at TBCR-affiliated institutions. This study was approved by the Nagoya University Graduate School of Medicine Ethics Committee (Approval No.: 2016-0388-3). Patients received IV-ABA infusions three times with 2-week intervals between infusions, and thereafter at 4-week intervals, according to drug labels and the Japan College of Rheumatology guidelines for treatment. Patients received a fixed dose of ABA at roughly 10 mg/kg body weight; patients weighing < 60 kg received 500 mg of ABA, those weighing 60–100 kg received 750 mg, and those weighing > 100 kg received 1000 mg^6^.

### Data collection

The following demographic data were recorded at initiation of treatment (baseline, week 0): age, sex, disease duration, rheumatoid factor (RF) positivity (≥ 20 IU/mL), history and number of previous bDMARDs, and concomitant treatment [methotrexate (MTX) and prednisolone (PSL)]. The following disease parameters were recorded at baseline and after 4, 12, 24, and 52 weeks of treatment: tender joint count (TJC) and swollen joint count (SJC) on 28 joints, patient and physician global assessment (PtGA and PhGA, respectively) of disease activity, modified health assessment questionnaire (mHAQ) score^18,19^, serum c-reactive protein (CRP) levels, erythrocyte sedimentation rate (ESR), and matrix metalloproteinase-3 (MMP-3) levels. Disease activity was evaluated at baseline and 4, 12, 24, and 52 weeks using the simplified disease activity index (SDAI), which includes data from the above-mentioned disease parameters. Radiographs of bilateral hands/wrists and feet at baseline and at 52 weeks were available for 171 patients. The timing of X-ray photography was allowed if within plus or minus three months. Images were scored using van der Heijde modified Sharp method independently by two trained readers^20^.

### Category of disease activity and radiographic remission

Disease activity was categorized as follows: remission (SDAI ≤ 3.3), low disease activity (LDA; 3.3 < SDAI ≤ 11), moderate disease activity (MDA; 11 < SDAI ≤ 26), and high disease activity (HDA; SDAI > 26). Radiographic remission was defined by a change in the modified total Sharp score (mTSS) ≤ 0.5 from baseline to 52 weeks^21–23^. We primarily evaluated the proportion of patients who achieved LDA at 52 weeks and of patients who achieved radiographic remission at 52 weeks.

### Statistical analysis

Demographic and disease characteristics are reported using descriptive statistics. All results are expressed as mean ± standard deviation (SD) or percentage (%). Student’s t test was used for 2-group comparisons, and the chi-square test for categorical variables. The last observation carried forward (LOCF) method was used in each analysis.

Multivariate analysis (logistic regression) was performed to identify factors that predict the achievement of LDA at 52 weeks. We assessed age, sex, disease duration, previous history of bDMARDs, concomitant MTX, concomitant PSL, SDAI at baseline, mHAQ score at baseline, and ACPA positivity. Adjusted odds ratios (ORs) with 95% confidence intervals (CIs) were calculated after adjusting for all variables.

All statistical tests were two-tailed, with significance defined as *p* < 0.05. All analyses were performed with SAS version 9.4 software (SAS Institute Inc., Cary, NC, USA).

## Results

### Patient characteristics

In total, 554 patients were enrolled in this study. We compared clinical disease activity and radiographic progression between ACPA-negative [ACPA (−)] and ACPA-positive [ACPA (+)] groups. Patient characteristics at baseline were comparable between these two groups except for mean value of ACPA and proportion of RF positivity (Table 1).

**Table 1:** Comparisons of baseline characteristics between ACPA-negative [ACPA (−)] and ACPA-positive [ACPA (+)] groups.

N	ACPA (−)	ACPA (+)	*p* value
	107	447	
Age (years)	67.3 ± 13.7	68.0 ± 11.3	0.583
Sex (% female)	82.2	79.0	0.451
Disease duration (years)	10.4 ± 10.1	12.5 ± 11.9	0.082
Body weight (kg)	51.9 ± 11.4	51.1 ± 9.1	0.412
ACPA (U/mL)	4.5 ± 4.2	370.8 ± 545.1	< 0.001
RF positive (%)	44.6	82.7	< 0.001
eGFR	71.8 ± 24.9	73.4 ± 25.0	0.547
KL-6 (U/mL)	308.6 ± 263.9	339.3 ± 272.6	0.367
Concomitant MTX use (%)	49.0	41.2	0.162
MTX dose (mg/week)^a^	9.0 ± 3.0	8.6 ± 3.5	0.550
Oral PSL use (%)	44.9	52.9	0.160
Oral PSL dose (mg/day)^a^	5.6 ± 3.1	5.5 ± 3.3	0.881
Previous biologics (%)	68.2	72.0	0.434
No. previous biologics^a^	1.5 ± 0.9	1.6 ± 0.8	0.813
**SDAI**	20.8 ± 14.0	22.2 ± 12.7	0.381
TJC, 0–28	5.6 ± 5.7	5.5 ± 5.2	0.796
SJC, 0–28	4.5 ± 4.6	4.9 ± 4.7	0.485
PtGA, 0–10 cm	4.9 ± 2.8	5.2 ± 2.6	0.238
PhGA, 0–10 cm	4.1 ± 2.6	4.2 ± 2.2	0.682
CRP (mg/dL)	2.0 ± 2.6	2.4 ± 3.9	0.326
MMP-3 (ng/mL)	191.9 ± 243.2	190.8 ± 200.8	0.966
mHAQ	0.85 ± 0.71	0.83 ± 0.68	0.860
mTSS	64.0 ± 65.8	71.8 ± 87.7	0.631

*ACPA* anti-citrullinated protein/peptide antibody, *RF* rheumatoid factor, *eGFR* estimated glomerular filtration rate, *KL-6* Krebs von den Lungen-6, *MTX* methotrexate, *PSL* prednisolone, *SDAI* simplified disease activity index, *TJC* tender joint count, *SJC* swollen joint count, *PtGA* patient’s global assessment, *PhGA* physician’s global assessment, *CRP* C-reactive protein, *MMP-3* matrix metalloproteinase-3, *mHAQ* modified health assessment questionnaire, *mTSS* van der Heijde modified total Sharp score.

^a^Mean among patients receiving the drug.

### Changes in disease activity

**Figure 1:**
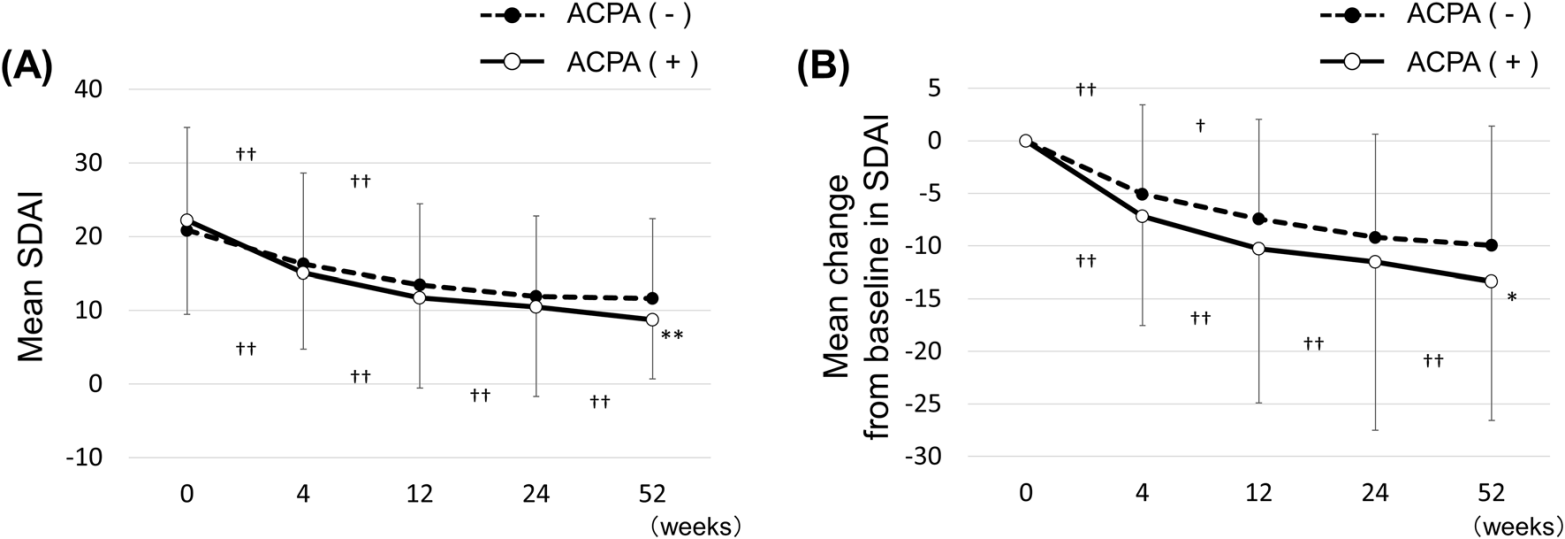
Comparisons of disease activity between ACPA-negative [ACPA (−)] and ACPA-positive [ACPA (+)] groups. (**A**) Transition of mean SDAI score. (**B**) Mean change from baseline in SDAI score. *ACPA* anti-citrullinated protein/peptide antibody, *SDAI* simplified disease activity index. *p < 0.05, **p < 0.01, Student’s t-test, compared with ACPA (−) group. ^†^*p* < 0.05, ^††^*p* < 0.01, paired t-test, comparisons between each time point.

Mean SDAI score decreased significantly, from 22.2 ± 12.7 at baseline to 8.7 ± 8.1 at 52 weeks in the ACPA (+) group, and from 20.8 ± 14.0 to 11.6 ± 10.8 in the ACPA (−) group. We also observed a significant difference between groups at 52 weeks (*p* = 0.0029), but no difference from baseline to 24 weeks (Fig. 1A). Change in SDAI score from baseline (ΔSDAI) at 52 weeks was significantly greater in the ACPA (+) group (− 13.4 ± 13.2 vs − 9.9 ± 11.3, *p* = 0.0265), while no difference was observed between groups from baseline to 24 weeks (Fig. 1B). Mean SDAI score and mean ΔSDAI score consistently decreased to 52 weeks in the ACPA (+) group, while statistical significance was observed only at 0–4 and 4–12 weeks in the ACPA (−) group.

**Figure 2:**
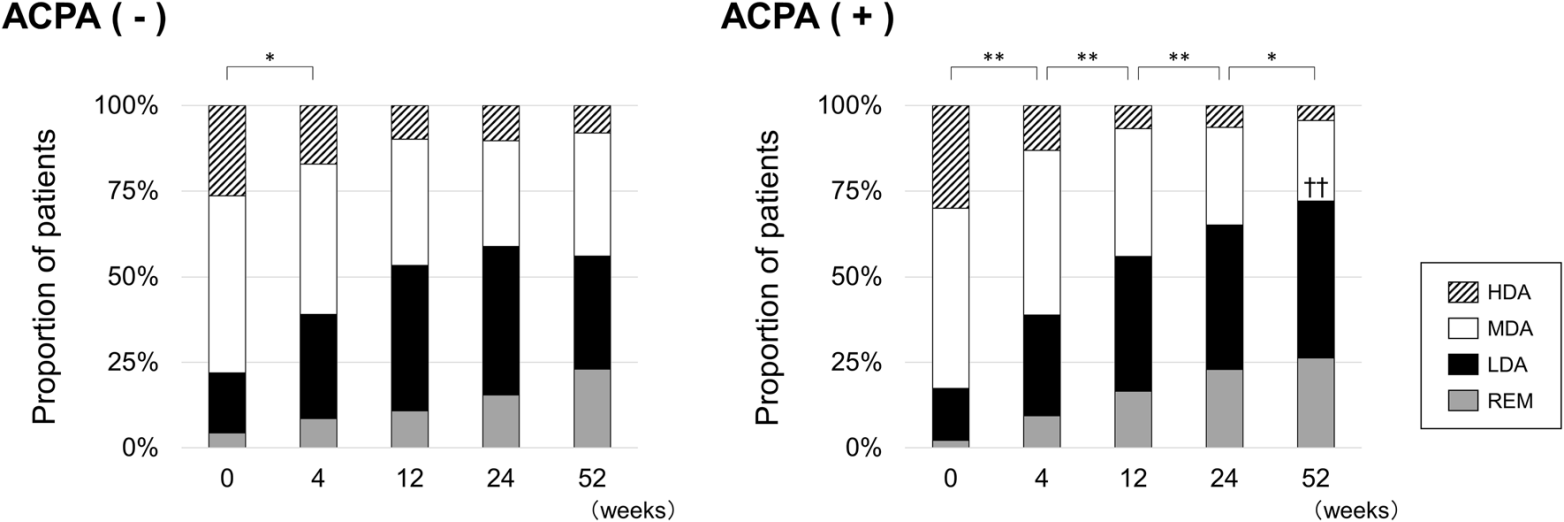
Categorical distribution of disease activity defined by SDAI score in ACPA-negative [ACPA (−)] and ACPA-positive [ACPA (+)] groups. *ACPA* anti-citrullinated protein/peptide antibody, *SDAI* simplified disease activity index, *HDA* high disease activity, *MDA* moderate disease activity, *LDA* low disease activity, *REM* remission. *p < 0.05, **p < 0.01 in LDA achievement rate, chi-square test. ^††^*p* < 0.01 in LDA achievement rate, chi-square test, compared with ACPA (−) group.

Figure 2 shows the categorical distribution of disease activity defined by SDAI score. There was no significant difference in the proportion of patients who achieved LDA or remission between the ACPA (−) and ACPA (+) groups from baseline to 24 weeks. The ACPA (+) group showed a significantly higher achievement rate of LDA at 52 weeks compared to the ACPA (−) group (72.1 vs 56.0%, *p* = 0.0019). Additionally, the LDA achievement rate persistently increased to 52 weeks in the ACPA (+) group, while we observed a significant difference only between baseline and 4 weeks in the ACPA (−) group.

**Figure 3:**
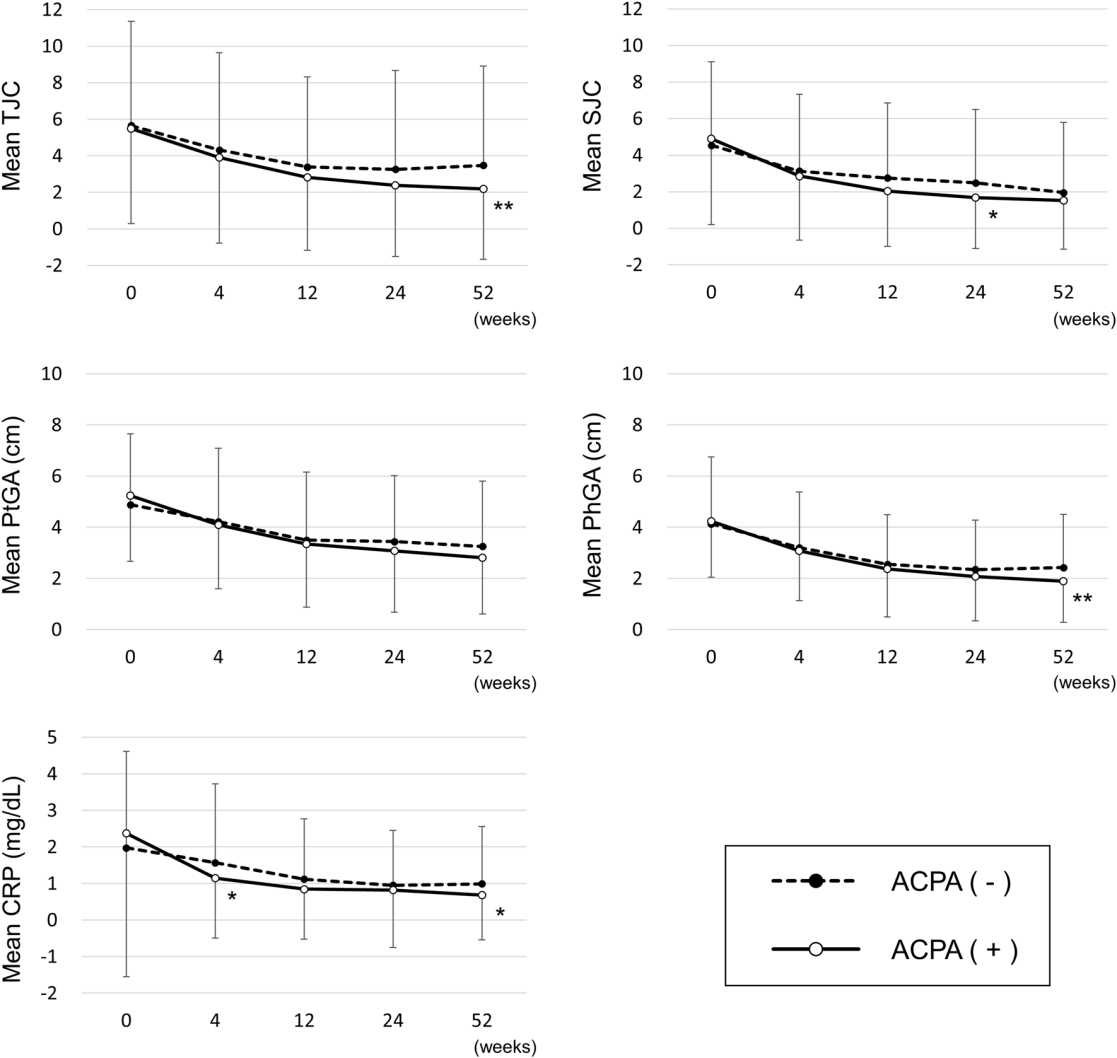
Comparisons of disease activity indices that are components of the SDAI score. *ACPA* anti-citrullinated protein/peptide antibody, *SDAI* simplified disease activity index, *TJC* tender joint count, *SJC* swollen joint count, *PtGA* patient’s global assessment, *PhGA* physician’s global assessment, *CRP* C-reactive protein. *p < 0.05, **p < 0.01, Student’s t-test, compared with ACPA (−) group at each time point.

We evaluated the change in each component of SDAI (Fig. 3). We observed a significant difference between the ACPA (−) and ACPA (+) groups at 52 weeks in TJC (3.5 ± 5.4 vs 2.2 ± 3.9, *p* = 0.0059), PhGA (2.4 ± 2.1 vs 1.9 ± 1.6, *p* = 0.0065), and CRP (1.00 ± 1.57 vs 0.68 ± 1.22, *p* = 0.0291).

### Rates of treatment retention and discontinuation due to inadequate response and adverse events

**Figure 4:**
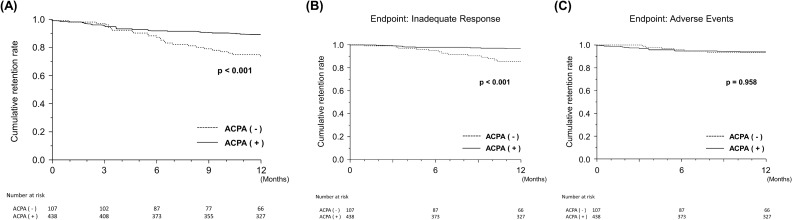
Comparisons of drug retention rate of abatacept estimated by Kaplan–Meier curves between ACPA-negative [ACPA (−)] and ACPA-positive [ACPA (+)] groups. (**A**) Overall drug retention rate. (**B**) Drug retention rate with discontinuation due to inadequate response as the endpoint. (**C**) Drug retention rate with discontinuation due to adverse events as the endpoint. *ACPA* anti-citrullinated protein/peptide antibody.

We found a significant difference in the retention rate of ABA treatment at 52 weeks between the ACPA (−) and ACPA (+) groups, as estimated by Kaplan–Meier analysis (73.8 vs 89.0%, *p* = 0.0002) (Fig. 4A). A significant difference was also observed in the discontinuation rate due to inadequate response at 52 weeks (14.4 vs 3.5%, *p* < 0.0001) (Fig. 4B), while no difference was observed in the discontinuation rate due to adverse events (6.5 vs 6.3%, *p* = 0.9582) (Fig. 4C).

### Factors predicting achievement of LDA at 52 weeks

Univariate and multivariate logistic regression analyses were performed to identify predictors of LDA achievement at 52 weeks. In the univariate logistic regression analysis, the following variables at baseline were found to be associated with LDA achievement at 52 weeks after ABA initiation: SDAI score, mHAQ score, and ACPA positivity (Table 2). The multivariate logistic regression analysis revealed the same three variables to be independently associated with LDA achievement at 52 weeks.

**Table 2:** Predictive factors for LDA achievement at 52 weeks.

Variables	Univariate		Multivariate	
	OR	*p* value	Adjusted OR	*p* value
Age	0.99 (0.98–1.01)	0.439	1.00 (0.97–1.02)	0.749
Male (vs. female)	1.12 (0.70–1.80)	0.634	0.79 (0.40–1.58)	0.511
Disease duration	0.99 (0.97–1.00)	0.053	0.99 (0.97–1.01)	0.468
Bio-naïve	1.23 (0.81–1.85)	0.335	1.18 (0.67–2.08)	0.575
MTX use	1.12 (0.75–1.69)	0.585	1.14 (0.66–1.95)	0.649
PSL use	0.82 (0.55–1.23)	0.329	0.97 (0.58–1.64)	0.923
SDAI at baseline	0.96 (0.94–0.97)	**< 0.001**	0.96 (0.94–0.98)	** < 0.001**
mHAQ	0.50 (0.36–0.69)	**< 0.001**	0.57 (0.38–0.86)	*0.008*
ACPA-positive	2.03 (1.29–3.17)	*0.002*	2.61 (1.36–5.00)	*0.004*

*LDA* low disease activity, *OR* odds ratio, *MTX* methotrexate, *PSL* prednisolone, *SDAI* simplified disease activity index, *mHAQ* modified health assessment questionnaire, *ACPA* anti-citrullinated protein/peptide antibody.

### Structural outcomes

**Figure 5:**
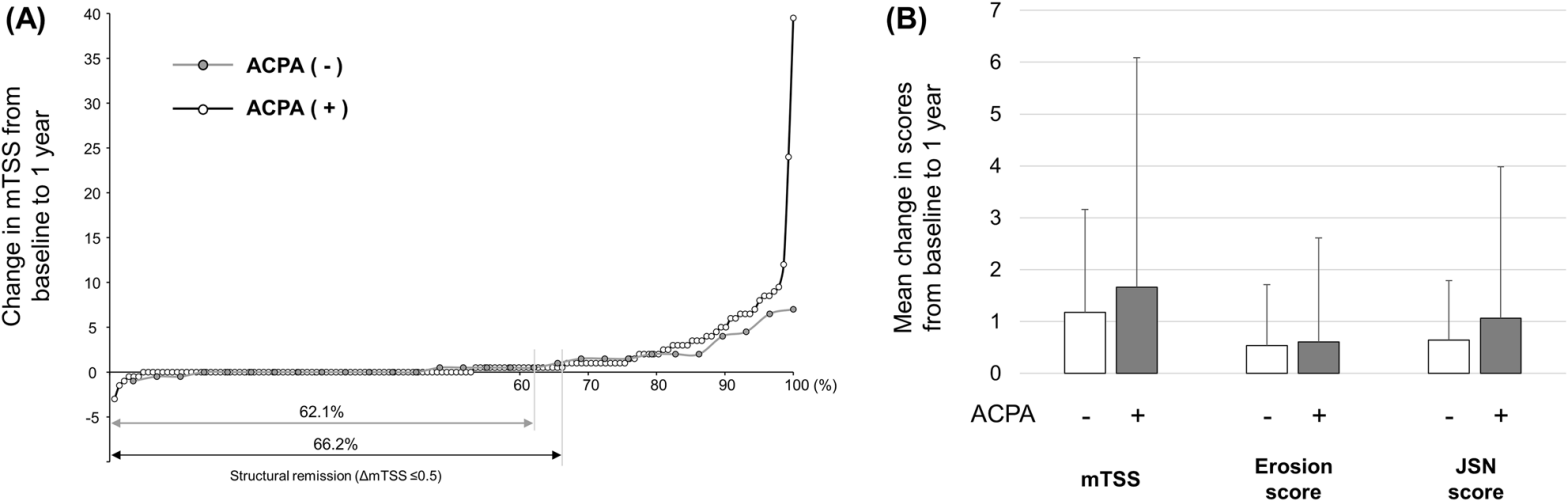
Comparisons of structural outcomes between ACPA-negative [ACPA (−)] and ACPA-positive [ACPA (+)] groups. (**A**) Cumulative probability plot of change from baseline to 52 weeks in van der Heijde modified total Sharp score (mTSS). (**B**) Mean value of mTSS, erosion score, and joint space narrowing (JSN) score.

Sequential radiographs of bilateral hands/wrists and feet at baseline and 52 weeks were obtained from 142 patients in the ACPA (+) group and 29 patients in the ACPA (−) group. The structural remission at 52 weeks, defined as a change in mTSS from baseline ≤ 0.5, was achieved in 94 patients (66.2%) in the ACPA (+) group and 18 patients (62.1%) in the ACPA (−) group, with no significant difference observed between groups (*p* = 0.670) (Fig. 5A). There was no significant difference in mean change from baseline to 52 weeks in mTSS (1.17 ± 1.98 vs 1.66 ± 4.42, *p* = 0.561), erosion score (0.53 ± 1.17 vs 0.60 ± 2.01, *p* = 0.861), and JSN score (0.638 ± 1.15 vs 1.06 ± 2.92, *p* = 0.446) between the ACPA (−) and ACPA (+) groups (Fig. 5B).

We found no significant difference in the baseline characteristics between the overall patients and the patients with sequential radiographs of bilateral hands/wrists and feet at baseline and 52 weeks (Table S1).

## Discussion

The main findings from this retrospective observational cohort study are as follows. The SDAI-LDA achievement rate at 52 weeks was significantly higher in the ACPA-positive group, and ACPA positivity was an independent predictor for LDA achievement. Interestingly, disease activity decreased consistently to 52 weeks in the ACPA-positive group, while statistically reached plateau by 12 weeks in the ACPA-negative group. The retention rate of ABA treatment was significantly higher in the ACPA-positive group. The achievement rates of radiographic remission at 52 weeks were similar between the ACPA-negative and ACPA-positive groups.

Consistent with previous reports^3,4,10,24^, we found that ACPA positivity was significantly associated with a good clinical response to ABA treatment also in Japanese patients who are genetically different from Caucasian. Another strength of our study was the novel demonstration of the detailed change in SDAI from baseline to 52 weeks in the ACPA-negative and ACPA-positive groups. We found that improvement in disease activity in the ACPA-negative group reached a plateau by 12 weeks while the disease activity continuously improved after 12 weeks to 52 weeks in the ACPA-positive group. We previously reported that 12 weeks was an adequate observational period for predicting the clinical efficacy of ABA at 52 weeks^7^. Especially in ACPA-negative patients, treatment adjustment should be considered if ABA effectiveness is judged to be clinically insufficient at 12 weeks.

Overall drug retention rate was significantly higher in the ACPA-positive group, consistent with previous reports. Gottenberg et al. reported a higher retention rate of ABA in ACPA-positive patients from the pan-European registry^25^. ACPA positivity was more frequent among patients still treated with ABA at 6 months compared to patients who discontinued ABA in the ORA registry^3^. Our investigation of retention rate by reasons for drug discontinuation revealed that the discontinuation rate due to adverse events was similar between the ACPA-negative and ACPA-positive groups, although we also noted a significant difference in discontinuation rate due to inadequate response.

Polyclonal ACPA isolated from the synovial fluid and peripheral blood of RA patients was reported to enhance osteoclast differentiation through a peptidylarginine deiminase (PAD)-dependent IL-8 neutralization^26^. Kleyer et al. reported that the structural bone damage started before the clinical onset of arthritis in subjects with ACPA positivity^27^. The association between ACPA positivity and joint destruction in RA patients has been reported from several groups. ACPA positivity was found to be an independent predictor for both formation of new bone erosion and cartilage destruction in RA patients participating in clinical trials of denosumab^12^. ACPA positivity was an independent predictor of joint destruction, even in RA patients in remission or LDA^28^. Thus, ACPA-positive RA patients are at high risk for joint destruction independently of disease activity. In our current results, the ACPA-positive group demonstrated a similar change in mTSS score to the ACPA-negative group, despite the significantly higher clinical response to ABA. We surmise that ABA treatment suppressed the progression of joint destruction in the high-risk group for joint destruction to the same extent as that in the low-risk group.

This study has several limitations. First, the sequential radiographic data at baseline and 52 weeks were obtained from only 30.1% of participants. The small number of samples may have been one of the reasons why there was no significant difference between the ACPA-positive and ACPA-negative group in the change in mTSS score. As this retrospective study was conducted in real clinical settings, we did not perform X-ray examinations in all patients, as would typically be done in a clinical trial. However, the current results regarding joint destruction may represent the overall cohort, since the patients with X-ray data had quite similar baseline characteristics compared to those in the overall patients. Additionally, the timing of X-ray photography was allowed if within plus or minus three months in this registry study. The varied timings of X-ray may have affected the results in this study. Second, the present study findings were all based on the use of IV ABA. However, the use of a subcutaneous (SC) formulation is currently widespread. Some patients may exhibit different responsiveness to SC and IV formulation. Further studies will be needed to determine whether similar results would be obtained with SC ABA as well.

## Conclusions

ABA treatment demonstrated a significantly higher clinical response and higher drug retention rate in ACPA-positive patients. Progression of joint destruction was similar between the ACPA-negative and ACPA-positive groups. Close attention should be paid to joint destruction even in patients showing a favorable response to ABA, especially when the ACPA status is positive.

## Supplementary information

Supplementary Table.
