# Supplementary material for: A retrospective analysis of the relationship between anti-cyclic citrullinated peptide antibody and the effectiveness of abatacept in rheumatoid arthritis patients
Source: Sci Rep. 2020 Nov 12;10:19717. doi: 10.1038/s41598-020-76842-4 (PMC7661716; doi:10.1038/s41598-020-76842-4)
Supplement: Supplementary file 1 — Supplementary Table. [file 41598_2020_76842_MOESM1_ESM.docx]

**Original Research Article**

**A retrospective analysis of the relationship between anti-cyclic citrullinated peptide antibody and the effectiveness of abatacept in rheumatoid arthritis patients**

Daihei Kida^1^, Nobunori Takahashi^2^, Atsushi Kaneko^1^, Yuji Hirano^3^, Takayoshi Fujibayashi^4^, Yasuhide Kanayama^5^, Masahiro Hanabayashi^6^, Yuichiro Yabe^7^, Hideki Takagi^8^, Takeshi Oguchi^9^, Takefumi Kato^10^, Koji Funahashi^11^, Takuya Matsumoto^12^, Masahiko Ando^13^, Yachiyo Kuwatsuka^13^, Eiichi Tanaka^14^, Hidekata Yasuoka^15^, Yuko Kaneko^16^, Shintaro Hirata^17^, Kosaku Murakami^18^, Yasumori Sobue^1^, Tsuyoshi Nishiume^1^, Mochihito Suzuki^1^, Yutaka Yokota^1^, Kenya Terabe^1^, Shuji Asai^1^, Naoki Ishiguro^1^, and Toshihisa Kojima^1^

(1) Department of Orthopedic Surgery and Rheumatology, Nagoya Medical Center, 4-1-1 Sanno-maru, Naka-ku, Nagoya, Aichi, Japan

(2) Department of Orthopedic Surgery and Rheumatology, Nagoya University Graduate School of Medicine, 65 Tsurumai-cho, Showa-ku, Nagoya, Aichi, Japan

(3) Department of Rheumatology, Toyohashi Municipal Hospital, 50 Hakken-nishi, Aotake-cho, Toyohashi, Japan

(4) Department of Orthopedic Surgery, Konan Kosei Hospital, 137 Oomatsubara, Takaya-cho, Konan, Aichi, Japan

(5) Department of Orthopedic Surgery, Toyota Kosei Hospital, 500-1 Ibohara, Josui-cho, Toyota, Aichi, Japan

(6) Department of Orthopedic Surgery, Ichinomiya Municipal Hospital, 2-2-22 Bunkyo, Ichinomiya, Aichi, Japan

(7) Department of Rheumatology, Tokyo Shinjuku Medical Center, 5-1 Tsukudo-cho, Shinjuku-ku, Tokyo, Japan

(8) Department of Orthopedic Surgery, Nagoya Central Hospital, 3-7-7 Taiko, Nakamura-ku, Nagoya, Aichi, Japan

(9) Department of Orthopedic Surgery, Anjo Kosei Hospital, 28 Higashihirokute, Anjo, Aichi, Japan

(10) Kato Orthopedic Clinic, 8-4 Minami-myoudaiji-cho, Okazaki, Aichi, Japan

(11) Department of Orthopedic Surgery, Kariya-Toyota General Hospital, 5-15 Sumiyoshi-cho, Kariya, Aichi, Japan

(12) Department of Orthopedic Surgery, Shizuoka Kosei Hospital, 23 Kitaban-cho, Aoi-ku, Shizuoka, Japan

(13) Department of Advanced Medicine, Nagoya University Hospital, 65 Tsurumai-cho, Showa-ku, Nagoya, Aichi, Japan

(14) Department of Rheumatology, School of Medicine, Tokyo Women’s Medical University, 8-1 Kawada-cho, Shinjuku-ku, Tokyo, Japan

(15) Division of Rheumatology, Department of Internal Medicine, Fujita Health University School of Medicine, 1-98 Dengakugakubo, Kutsukake-cho, Toyoake, Aichi, Japan

(16) Department of Internal Medicine, Keio University Hospital, 35 Shinanomachi, Shinjuku-ku, Tokyo, Japan

(17) Department of Clinical Immunology and Rheumatology, Hiroshima University Hospital, 1-2-3 Kasumi, Minami-ku, Hiroshima, Japan

(18) Department of Rheumatology and Clinical Immunology, Kyoto University Graduate School of Medicine, Kyoto, 54 Kawaharacho, Shogoin, Sakyo-ku, Kyoto, Japan

**Supplemental data**

**Table S1.** Comparisons of baseline characteristics between overall patients and patients with sequential radiographs of bilateral hands/wrists and feet at baseline and 52 weeks.

|  | **Overall** | **Pts with X-ray** |  |
| --- | --- | --- | --- |
| N | 554 | 171 |  |
|  |  |  | p-value |
| Age (years) | 67.9±11.81 | 68.6±9.99 | 0.482 |
| Sex (% female) | 79.60 | 78.95 | 0.853 |
| Disease duration (years) | 12.12±11.55 | 13.36±12.83 | 0.233 |
| Body weight (kg) | 51.24±9.59 | 51.5±9.85 | 0.759 |
| ACPA (U/mL) | 300.03±510.47 | 377.9±730.76 | 0.119 |
| RF positive (%) | 75.4 | 78.23 | 0.487 |
| eGFR | 73.11±24.92 | 73.52±24.37 | 0.851 |
| KL-6 (U/ml) | 333.7±270.98 | 345.8±277.93 | 0.642 |
| Concomitant MTX use (%) | 42.74 | 42.07 | 0.886 |
| MTX dose (mg/week) ^a^ | 8.7±3.36 | 9.02±3.28 | 0.522 |
| Oral PSL use (%) | 51.24 | 53.79 | 0.589 |
| Oral PSL dose (mg/day)^a^ | 5.51±3.27 | 5.91±3.97 | 0.375 |
| Previous biologics (%) | 71.3 | 73.68 | 0.544 |
| No. previous biologics^a^ | 0.45±0.84 | 0.42±0.85 | 0.659 |
| SDAI | 21.92±12.96 | 22.31±12.89 | 0.739 |
| TJC, 0-28 | 5.52±5.3 | 5.78±5.55 | 0.586 |
| SJC, 0-28 | 4.84±4.68 | 5.21±5.37 | 0.390 |
| PtGA, 0-10 cm | 51.66±26.05 | 51.45±26.76 | 0.928 |
| PhGA, 0-10 cm | 42.18±22.79 | 42.09±21.97 | 0.967 |
| CRP (mg/dL) | 2.3±3.72 | 2.18±3.14 | 0.699 |
| MMP-3 (ng/mL) | 243.69±261.04 | 223.59±205.91 | 0.368 |
| mHAQ | 0.83±0.69 | 0.8±0.61 | 0.549 |
| mTSS | 70.47±84.29 | 72.35±85.52 | 0.833 |

Pts: patients, ACPA: anti-citrullinated protein/peptide antibody, RF: rheumatoid factor, eGFR: estimated glomerular filtration rate, KL-6: Krebs von den Lungen-6, MTX: methotrexate, PSL: prednisolone, SDAI: simplified disease activity index, TJC: tender joint count, SJC: swollen joint count, PtGA: patient’s global assessment, PhGA: physician’s global assessment, CRP: C-reactive protein, MMP-3: matrix metalloproteinase-3, mHAQ: modified health assessment questionnaire, mTSS: van der Heijde modified total Sharp score.

^a^ Mean among patients receiving the drug
